# Evaluating electrochemical impedance spectroscopy for SEI characterization in lithium- and sodium-metal batteries

**DOI:** 10.1039/d6fd00049e

**Published:** 2026-04-24

**Authors:** Mohammed A. Zabara, Jeongjae Lee, Svetlana Menkin, Clare P. Grey

**Affiliations:** a Yusuf Hamied Department of Chemistry, University of Cambridge Lensfield Road Cambridge CB2 1EW UK; b The Faraday Institution, Quad One, Harwell Science and Innovation Campus Didcot OX11 ORA UK

## Abstract

Electrochemical impedance spectroscopy (EIS) is widely used to probe the solid electrolyte interphase (SEI) in alkali-metal batteries, yet the interpretation of impedance responses remains challenging due to the simultaneous influence of interfacial transport properties and evolving electrode morphology. Here, we systematically evaluate the capability of EIS to characterise SEI behaviour in symmetric lithium- and sodium-metal cells using carbonate and ether electrolytes. Time-resolved impedance measurements were combined with distribution-of-relaxation-times analysis, equivalent-circuit modelling, temperature-dependent EIS, and *in situ* NMR spectroscopy to track interfacial evolution during formation, cycling, and rest. EIS captures key interfacial changes, including resistance growth during open-circuit conditions and capacitance increases associated with expansion of the electrochemically active surface area during cycling. However, comparison with *in situ* NMR reveals that continuous microstructure formation and SEI accumulation can occur without being directly reflected in impedance-derived SEI parameters. Temperature-dependent analysis further shows that activation energies derived from resistance may include geometric contributions associated with surface-area changes, whereas analysis of the interfacial time constant provides a more surface-area-independent metric of SEI transport. These results demonstrate that while EIS is a powerful tool for monitoring interfacial evolution, impedance responses in metal-electrode systems must be interpreted carefully and supported by complementary techniques to distinguish intrinsic SEI properties from morphology-driven effects.

## Abstract 2

EIS tracks SEI evolution in Li/Na metal cells. However, combined with *in situ* NMR, results show impedance response is masked by the ongoing microstructural growth resulting in high surface area changes, requiring careful, multi-technique interpretation.

## Graphical abstract

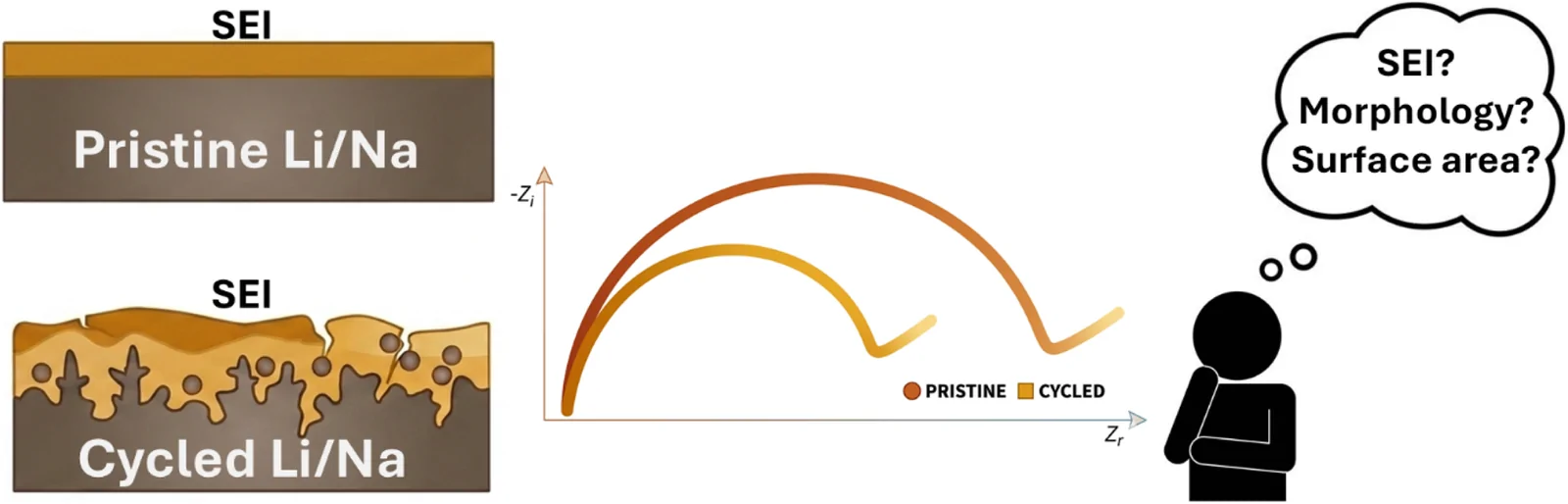

## Introduction

Metallic lithium and sodium anodes remain central to the pursuit of next-generation rechargeable batteries owing to their unmatched theoretical capacities and low redox potentials.^1,2^ While lithium metal has been studied extensively, sodium metal has recently re-emerged as a compelling alternative due to elemental abundance and potential cost advantages. Despite these differences, both systems share a critical and unresolved challenge: the formation of a solid electrolyte interphase (SEI) that is chemically heterogeneous, mechanically fragile, and dynamically evolving under electrochemical operation. The properties of the SEI control the redox process on the electrode (plating/stripping) and is critical to increase the reversibility and cycling efficiency of the cell.^3,4^

Electrochemical impedance spectroscopy (EIS) is routinely employed to probe SEI properties in both Li- and Na-metal batteries. However, its application to metallic anodes is frequently treated as a direct extension of methodologies developed for intercalation electrodes with similar assumptions for analysing the EIS data despite the fundamentally different natures between the two systems. Subsequently, in the literature, impedance features are commonly assigned to SEI resistance, charge-transfer resistance, or ion diffusion through surface films, yet such assignments vary widely even for nominally similar systems.^5–8^ This lack of consensus is particularly pronounced when comparing Li- and Na-metal electrodes, where impedance responses often differ substantially despite comparable experimental protocols.^9–11^

In contrast to intercalation electrodes with constant surface areas as defined by crystallite sizes (*e.g.*, graphite and hard carbon),^12,13^ metallic Li and Na electrodes undergo substantial morphological evolution during plating and stripping. A central complication specific to metallic electrodes is the dynamic evolution of real electrochemical surface area during plating and stripping: dendritic growth, roughening, and partial dissolution substantially increase the effective reaction area, while impedance values are commonly normalized to geometric area. Consequently, reductions in apparent SEI or charge-transfer resistance may arise from surface area expansion rather than genuine improvements in SEI ionic conductivity or stability. The real electrochemical surface area (ECSA) frequently diverges from the nominal geometric area due to dendritic growth, mossy deposits, pitting, and uneven dissolution.^14,15^ These surface changes alter local current distributions and effective reaction area, complicating the direct assignment of impedance elements to intrinsic SEI properties. A growing body of literature has emphasized that measured impedance changes often reflect surface area evolution rather than—or in addition to—changes in SEI composition or resistivity.^16–19^

Detailed impedance studies on Li-metal symmetric cells have demonstrated that multiple overlapping interfacial processes contribute to the mid-frequency response and that physically constrained circuit selection is essential to avoid over-interpretation of SEI elements.^19^ At the same time, comprehensive analyses of Li-metal SEI formation have emphasized that impedance-derived parameters inherently reflect a convolution of chemistry, morphology, and transport pathways rather than a single intrinsic material property.^20^ These challenges are further amplified for Na metal, where the SEI is typically more porous and chemically labile, leading to continuously evolving impedance signatures and less distinct semicircular features.^21^*Operando* impedance studies correlating morphology evolution with spectral changes have confirmed that mid-frequency features are strongly modulated by deposit topology and current distribution effects.^22^

Despite recent advances, important challenges remain in interpreting the SEI behaviour of metallic Li and Na electrodes using EIS. In particular, distinguishing intrinsic interphase properties from changes caused by evolving electrode morphology remains difficult. Monitoring the interface under open-circuit voltage (OCV) after plating and stripping is critical, as freshly deposited Li or Na can continue reacting chemically with electrolyte components after current interruption. As a result, impedance variations may reflect ongoing SEI growth or reconstruction rather than purely electrochemically driven processes. Moreover, changes in electrochemically active surface area during cycling can strongly influence the measured resistance and capacitance, potentially masking variations associated with SEI composition or thickness. These challenges highlight the need for systematic approaches that track impedance evolution together with independent probes of interfacial structure and chemistry. Establishing such correlations is essential for decoupling intrinsic SEI transport properties from morphology-driven effects and for enabling reliable interpretation of EIS responses in alkali-metal battery systems.

To address these challenges, we critically reassess the use of EIS for probing the SEI on metallic Li and Na electrodes in light of the dynamic and morphology-dependent nature of these interfaces. In particular, we focus on two factors which are difficult to deconvolute with only EIS data: (i) the continuously evolving structure of metallic electrodes during plating and stripping, and (ii) the associated evolution of electrochemically active surface area, which complicates quantitative interpretation of impedance magnitudes. To decouple these compounding factors, we propose a correlative *in situ* methodology that combines EIS with *in situ* nuclear magnetic resonance (NMR) spectroscopy to simultaneously track the chemical and morphological evolution of the microstructural metal surfaces. Temperature-dependent measurements are further employed to probe the kinetic aspects of the SEI processes captured by impedance and to evaluate how extracted activation energies are influenced by morphological evolution. By applying this integrated approach to symmetric Li‖Li and Na‖Na cells in both carbonate- and ether-based electrolytes, we deliberately examine interphases with contrasting structural characteristics. Carbonate electrolytes typically produce thicker, denser, and more heterogeneous SEI layers, whereas ether-based systems favour thinner and more porous interphases. This electrolyte-dependent contrast enables us to systematically evaluate how SEI thickness, heterogeneity, and porosity influence impedance features and their sensitivity to surface area evolution. Through this comparison across Li and Na systems, we propose a solution to deconvolute the chemistry-driven changes from morphology-driven changes in impedance data and establish a more rigorous, morphology-aware framework for interpreting impedance spectra of metallic electrodes.

## Methods

### Cell assembly

All sample preparation and cell assembly were performed in an argon-filled glovebox (O_2_ and H_2_O < 0.1 ppm). Alkali-metal symmetric cells (Li‖Li and Na‖Na) were fabricated using high-purity lithium and sodium 16-mm metal disc electrodes (Cambridge energy solutions). Standard CR2032 coin cells were used for electrochemical testing, while customized, non-magnetic casing designs (PEEK (polyether ether ketone) capsule cell) were utilized for the NMR measurements to prevent radio-frequency (RF) shielding.

### Electrolyte systems

For each metal, two distinct electrolyte systems were prepared: a carbonate-based electrolyte and an ether-based electrolyte. The carbonate electrolyte for lithium cells consisted of 1.0 M LiPF_6_ in a 3 : 7 volume ratio of ethylene carbonate (EC) and ethyl methyl carbonate (EMC) (Solvionic) used without further procedures, and for sodium cells, 1.0 M NaPF_6_ dissolved in a 1 : 1 volume ratio of EC and diethyl carbonate (DEC). Homemade NaPF_6_ was used in this study.^23^ The EC:DEC mixture was prepared from EC (Sigma-Aldrich, battery grade, ≥99%, acid <10 ppm, H_2_O < 10 ppm) and DEC (Sigma-Aldrich, battery grade, ≥99%, acid <10 ppm, H_2_O < 10 ppm), which was further dried using molecular sieves, and tested by Karl-Fischer titration, which gave a result of less than 30 ppm H_2_O. The ether-based electrolyte comprised 1.0 M lithium bis(trifluoromethanesulfonyl)imide (LiTFSI) (Sigma-Aldrich) or 1.0 M NaPF_6_ dissolved in a 1 : 1 volume ratio of 1,3-dioxolane (DOL) and 1,2-dimethoxyethane (DME).

### Electrochemical measurements

Galvanostatic plating and stripping were conducted using a multichannel potentiostat/galvanostat (Biologic VSP-300). Symmetric cells were subjected to continuous cycling with the current density stepwise increased from 0.3 mA cm^−2^ to 1.0 mA cm^−2^, applying a fixed areal capacity for each cycle.

Time-resolved EIS was performed continuously at specific resting intervals following the plating/stripping steps. Impedance spectra were recorded over a frequency range from 1 MHz to 1 Hz with an alternating-current (AC) voltage amplitude of 5 mV.

For the temperature-dependent impedance measurements, the cells were placed in an environmental chamber. EIS data were collected across a temperature range of 15 °C to 45 °C (in 10 °C increments). A minimum thermal equilibration time of 30 minutes was enforced at each step. The resulting EIS data were analysed using equivalent circuit modelling in Gamry Echem Analyst and Distribution of Relaxation Times (DRT) analysis using RelaxIS 3 software.

### *In situ* NMR measurements

The structural evolution of the alkali-metal deposits was monitored using *in situ* Nuclear Magnetic Resonance (NMR) spectroscopy. Measurements were performed on a Bruker 7.0 T Avance NEO spectrometer operating at ^7^Li and ^23^Na Larmor frequencies of 116.6 and 79.4 MHz, respectively, using an *in situ* electrochemical probe (NMR Services GmbH, Erfurt, Germany). Measurements were performed with the battery stack oriented parallel to the applied magnetic field inside the probe. To capture the morphological changes in real time, spectra were continuously acquired during the galvanostatic cycling protocols. A single-pulse excitation sequence with recycle delays of 1s was used for all spectra. RF powers of 26 and 32 kHz were used for ^7^Li and ^23^Na measurements, respectively. The spectra were referenced externally to 1.0 M aqueous solutions of LiCl and NaCl (0 ppm).

## Results and discussion

Fig. 1 compares the EIS response (1 MHz–1 Hz) at OCV of symmetric Li‖Li and Na‖Na cells employing 1 M LiPF_6_ in EC:EMC, 1 M LiTFSI in DOL:DME, 1M NaPF_6_ in EC:DEC, and 1M NaPF_6_ in DOL:DME evaluated in both pristine and cycled states. The combination of Nyquist analysis and DRT deconvolution enables identification of the SEI-related processes. A pronounced electrolyte- and metal-dependent evolution of the impedance response is observed for both pristine and cycled states. In all systems, the overall impedance magnitude decreases after cycling; however, this reduction is significantly more pronounced for Li-based cells, whereas Na systems exhibit comparatively modest changes. DRT analysis reveals a dominant relaxation process in the pristine state across all electrolytes, which can be attributed to SEI-associated ion transport. Upon cycling, this process persists but shifts toward shorter time constants in Li systems, indicating accelerated interfacial kinetics or increased effective surface area, while Na systems display close to no shift in relaxation time, suggesting comparatively stable interfacial dynamics under the same conditions.

**Fig. 1:**
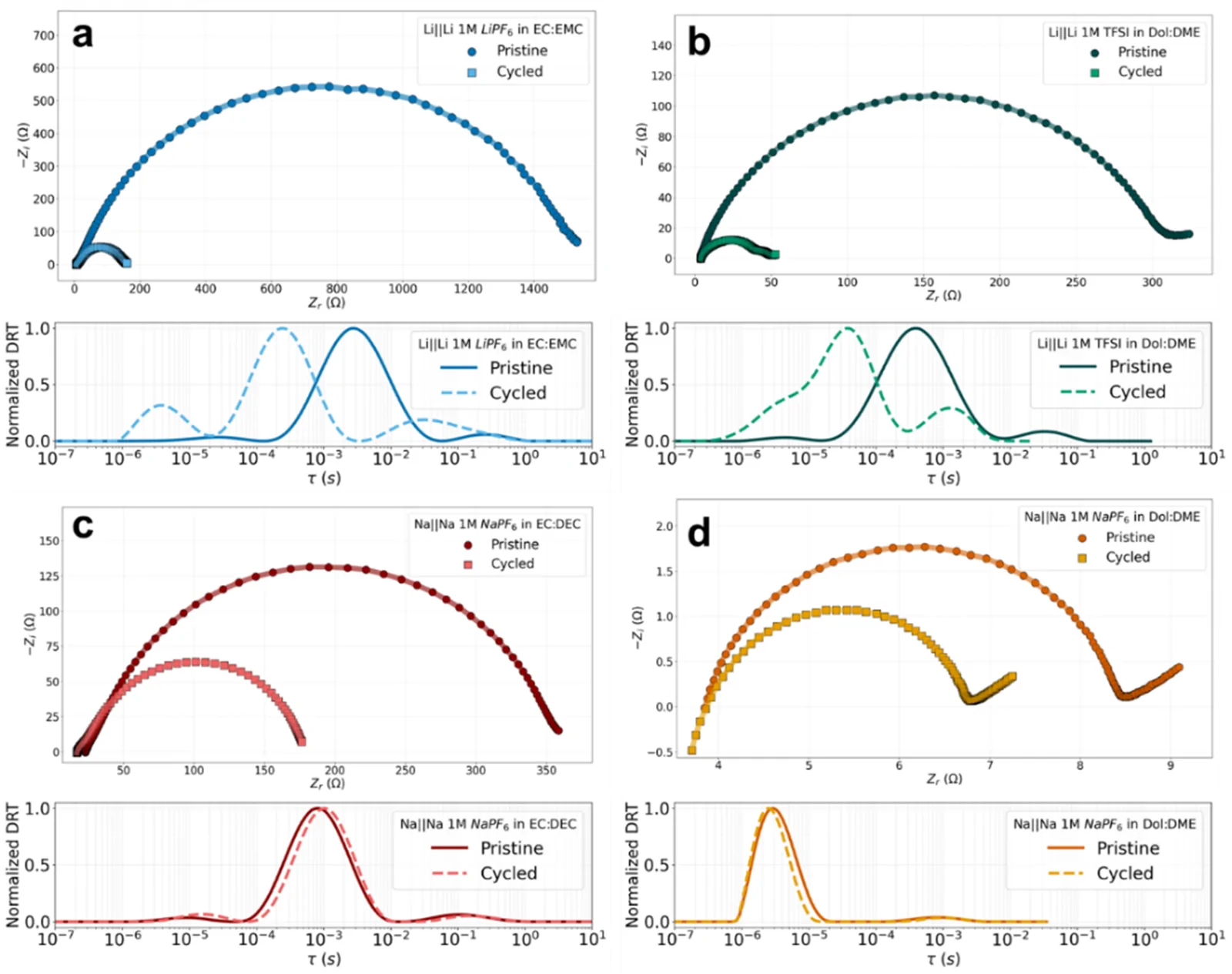
Nyquist (top) and DRT (bottom) plots of Li|Li and Na‖Na symmetric cells under different electrolytes for fresh and cycled conditions. (a) 1M LiPF6 in 3 : 7 EC:EMC, (b) 1M TFSI in 1 : 1 DOL:DME, (c) 1M NaPF6 in 1 : 1 EC:DEC, and (d) 1M NaPF6 in 1 : 1 DOL:DME.

In the EC:EMC electrolyte (Fig. 1a), the fresh Li‖Li cell exhibits a large depressed semicircle spanning a wide range along the real impedance axis. After cycling, the diameter of this semicircle decreases substantially. The corresponding DRT spectrum of the fresh cell reveals mainly a distinguishable relaxation feature between 1–10 ms with other small peaks at higher and lower time constants. Following cycling, the DRT profile changes in both intensity and position: the dominant relaxation shifts toward shorter time constants and its magnitude decreases, while the relative contributions of other relaxation features are more pronounced. These observations indicate a redistribution of interfacial kinetic processes upon cycling. However, from impedance data alone, it cannot be determined whether the decrease in semicircle diameter originates from changes in SEI composition, structural reconstruction of the interphase, or an increase in effective surface area caused by morphological evolution during repeated plating and stripping.

In the DOL:DME electrolyte, the pristine Li‖Li cell exhibits a markedly smaller semicircle compared to the EC:EMC system. The overall impedance magnitude is lower, and the DRT response is dominated by a dominant faster relaxation feature (0.1–1 ms). Other relaxation peaks are also observed at lower and higher time constants with lower peaks. Upon cycling, the changes in both Nyquist and DRT representations are comparable to the EC:EMC system. The principal relaxation shifts to faster relaxation and the relative difference in intensity to the other processes decreases. Relative to the carbonate system, two clear observational differences emerge: the absolute impedance magnitude is reduced, and the redistribution of relaxation times is faster. These differences demonstrate that electrolyte identity strongly influences the interfacial kinetic structure captured by EIS, which can be linked to the type of SEI formed due to the type of electrolyte (EC:EMC *vs.* DOL:DME).

Extending this analysis to symmetric Na‖Na cells under analogous electrolyte conditions demonstrates different results. In the EC:DEC electrolyte, the fresh Na cell exhibits a semicircle with a smaller real-axis extent than that observed for Li in the corresponding EC:EMC system. After cycling, the semicircle diameter decreases, although the reduction is much smaller than that observed in the Li system. The DRT spectrum is characterized primarily by a single dominant relaxation feature at 1 ms with limited secondary contributions. Cycling induces a small reduction in peak intensity and a slight shift in relaxation time to a slower time constant, the opposite of what was observed in the Li system. As with the Li system, this change may reflect alterations in the interphase itself or changes in the effective surface area resulting from morphology evolution during cycling. The impedance response alone does not allow these contributions to be separated.

In the DOL:DME electrolyte, the Na‖Na cell displays the lowest overall impedance among all systems studied. The Nyquist plot shows a compact semicircle, and the DRT spectrum is dominated by a sharp high-frequency relaxation with a time constant between 1–10 µs indicating the substantially higher ionic conductivity of the formed SEI compared to other systems. Notably, cycling produces only minor changes in both representations. The preservation of the relaxation structure suggests limited redistribution of kinetic processes during cycling.

Taken together, Fig. 1 demonstrates that EIS combined with DRT analysis resolves electrolyte-dependent and metal-dependent differences in SEI kinetic distributions. The measurements clearly show that (i) impedance magnitude varies substantially between carbonate and ether electrolytes, (ii) the number and breadth of relaxation features differ across systems, and (iii) cycling modifies both the magnitude and distribution of relaxation processes to varying degrees. However, as noted in the Introduction, the origin of these changes cannot be uniquely assigned based on impedance alone: the observed evolution may result from SEI compositional changes, structural reorganization, variations in interphase thickness, or changes in the effective surface area induced by morphological growth during plating and stripping. Thus, a methodology to allow decoupling the changes in microstructural surface area from SEI growth is required; in the following section, we show that this is possible using combined *in situ* NMR and EIS.

### EIS and *in situ* NMR analysis

Following the impedance trends presented in Fig. 1, we next investigate the dynamic evolution of the EIS response during galvanostatic cycling in direct correlation with *in situ* NMR measurements. For that we used the current profile shown in Fig. 2, which systematically subjects the symmetric cells to distinct operational regimes to isolate SEI formation, dynamic electrochemical stress, and relaxation. As denoted by the numbered indices (1–10), simultaneous EIS and corresponding NMR acquisitions were performed at specific intervals across the protocol all measured at OCV. The sequence begins with a baseline measurement of the pristine interface (point 1), followed by a low-current formation step at ±0.3 mA cm^−2^ (±0.3 mAh cm^−2^) designed to establish the initial passivating interphase. Following a subsequent 1 h rest period (points 2 and 3) to capture the rested SEI, the cells are subjected to rigorous, high-rate cycling at ±1.0 mA cm^−2^ (±0.5 mAh cm^−2^). Intermittent measurements (points 4–7) are recorded during these high-current cycles to track the dynamic morphological and interfacial transformations under operational stress. Finally, an extended post-cycling rest phase (points 8–10) enables the monitoring of structural relaxation and transient recovery processes. This structured protocol provides a comprehensive temporal map, allowing us to decouple the intrinsic interphase kinetics from the morphological evolution of the metal deposit across its entire lifecycle.

**Fig. 2:**
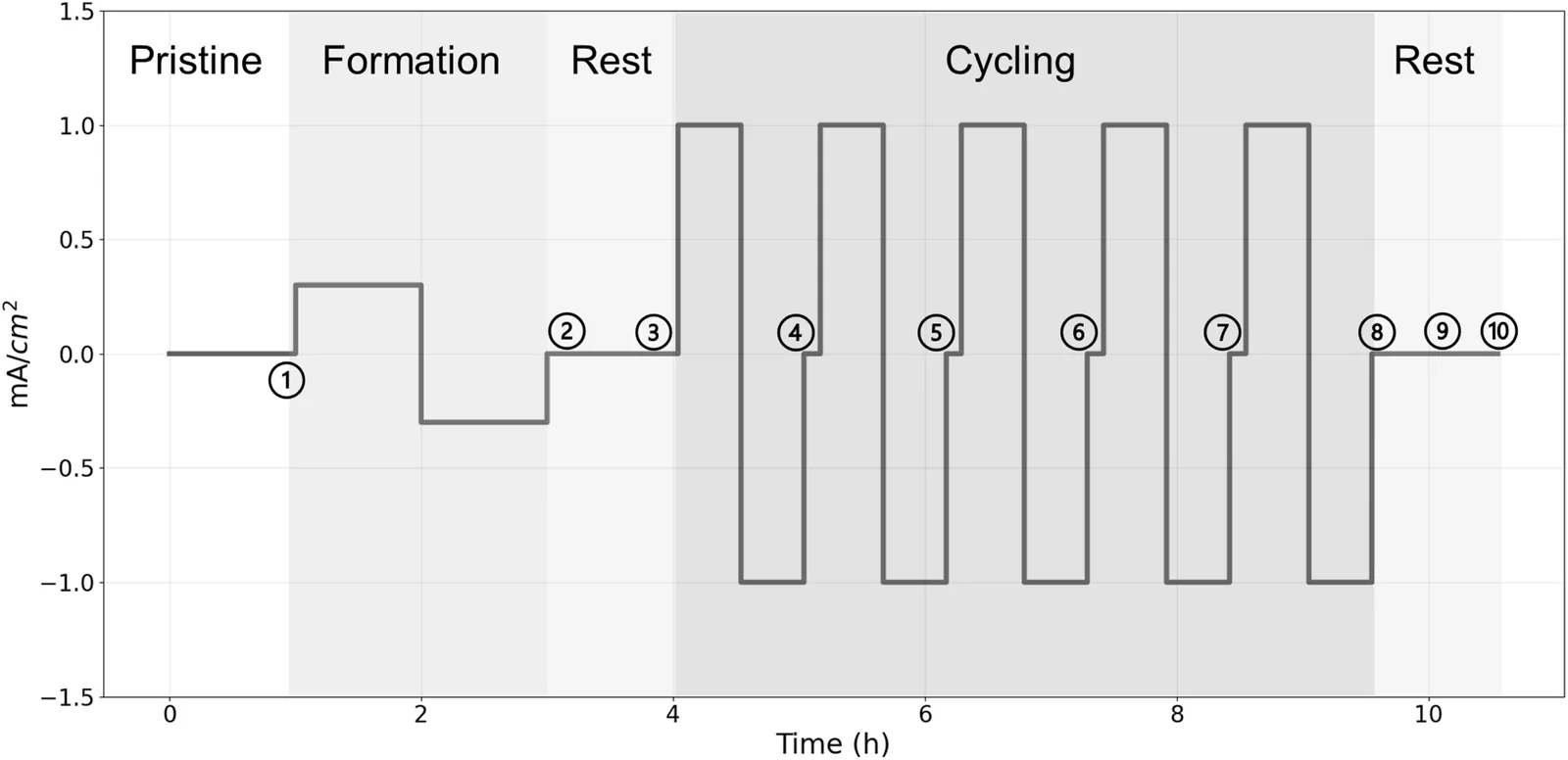
Galvanostatic cycling current profile used to study the time-resolved EIS and the corresponding NMR responses of the Li‖Li and Na‖Na symmetric cells. The numbers indicate the points at which the EIS measurements were taken at OCV.

Fig. 2 demonstrates the applied galvanostatic cycling current profile for the Li‖Li and the Na‖Na cells with the points where the EIS and NMR measurements were taken. The profile starts with the application of a small current density current that will result in the formation of the SEI and initiate the morphological changes in the surface of the metallic electrodes. It is then followed by a 1 h rest step to observe the change of the EIS response as no current is applied, which is followed by five cycling steps with relatively high current density that have rest steps between for the EIS measurements. Lastly, two EIS measurements are taken at the end of the cycling with 0.5 h rest periods.

Fig. 3 and 4 display the time-resolved EIS and NMR behavior of symmetric Li‖Li and Na‖Na cells, respectively. Each figure integrates the voltage profile, Nyquist spectra collected at defined intervals under OCV conditions, and the corresponding *in situ* NMR spectra acquired at the same states of cycling. This correlative approach enables direct comparison between impedance evolution and structural changes occurring at the electrode surface during repeated plating and stripping. The *in situ* NMR spectra of both Li‖Li and Na‖Na cells exhibit two distinct spectral regions. The first, located in the diamagnetic (∼0 ppm) range, contains contributions from the electrolyte (sharp liquid signal) and SEI (broad, due to unaveraged dipolar coupling in solids) species and thus provides insight into SEI evolution. The second, appearing at higher chemical shifts due to the Knight shift (hyperfine interaction of nucleus with spins of conduction electrons), corresponds to metallic Li (∼240 ppm) or Na (∼1100 ppm) and reflects the electronic environment of the metal. Changes in the metallic resonance—such as intensity variations and line-shape evolution—serve as indicators of morphological transformations and the relative abundance of micro-structured deposits formed during plating and stripping. Of note, metallic microstructures (mossy or dendritic features) typically show up as a high-frequency shoulder to the bulk-metal signal.^24,25^ By correlating SEI impedance features with the evolution of the metallic Li/Na NMR resonance, we establish a direct relationship between electrochemical kinetics and morphology-driven structural changes in the studied systems.

**Fig. 3:**
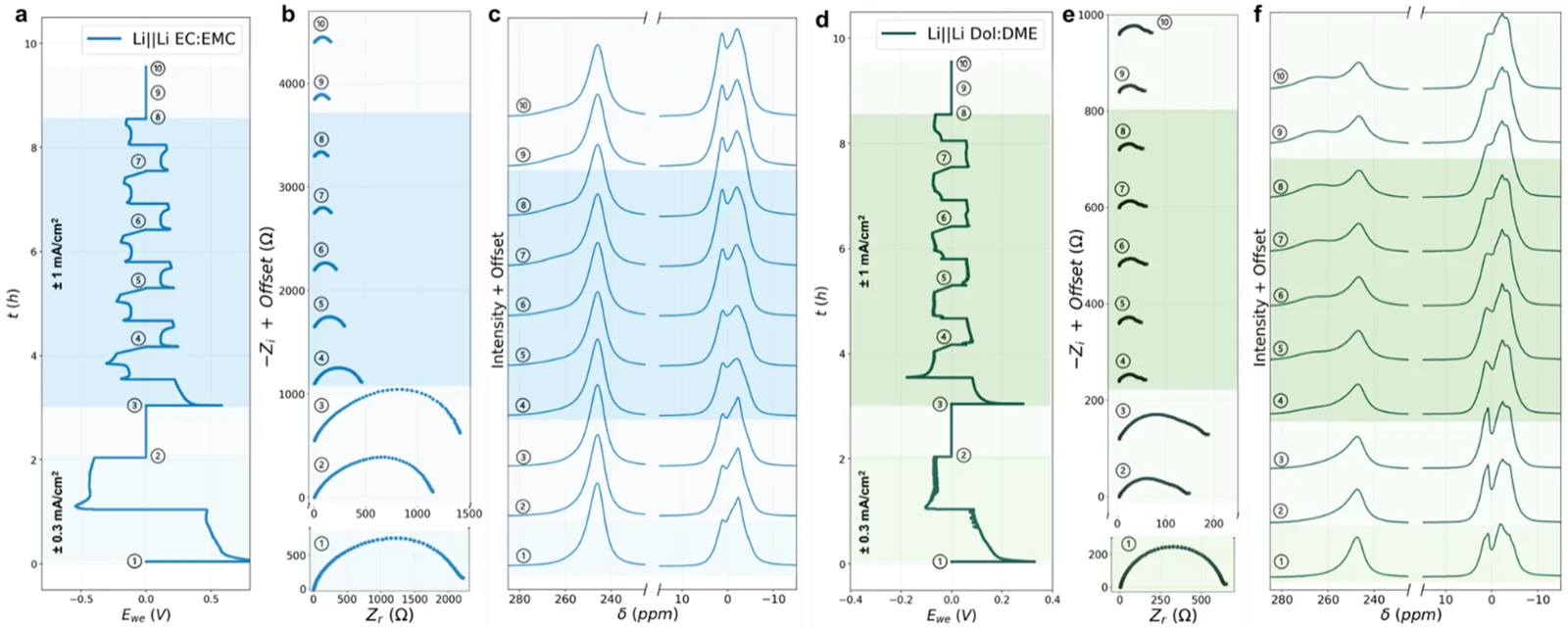
Time-resolved EIS and NMR spectroscopic evolution of symmetric Li‖Li cells in EC:EMC and DOL:DME electrolytes during galvanostatic cycling. (a and d) Voltage profiles recorded during stepwise galvanostatic cycling with an initial formation cycle at current densities of ±0.3 mA cm^−2^ (±0.3 mAh cm^−2^), followed by high-current-density cycles at ±1 mA cm^−2^ (±0.5 mAh cm^−2^) for Li‖Li cells in (a) EC:EMC and (d) DOL:DME. Numbered markers indicate the states at which impedance and spectroscopic measurements were collected. (b and e) Corresponding Nyquist plots obtained at the selected cycling stages, displayed with vertical offsets for clarity. (c and f) *In situ* NMR responses collected at the same states, shown as intensity (offset for clarity) *versus* chemical shift (*δ*, ppm).

**Fig. 4:**
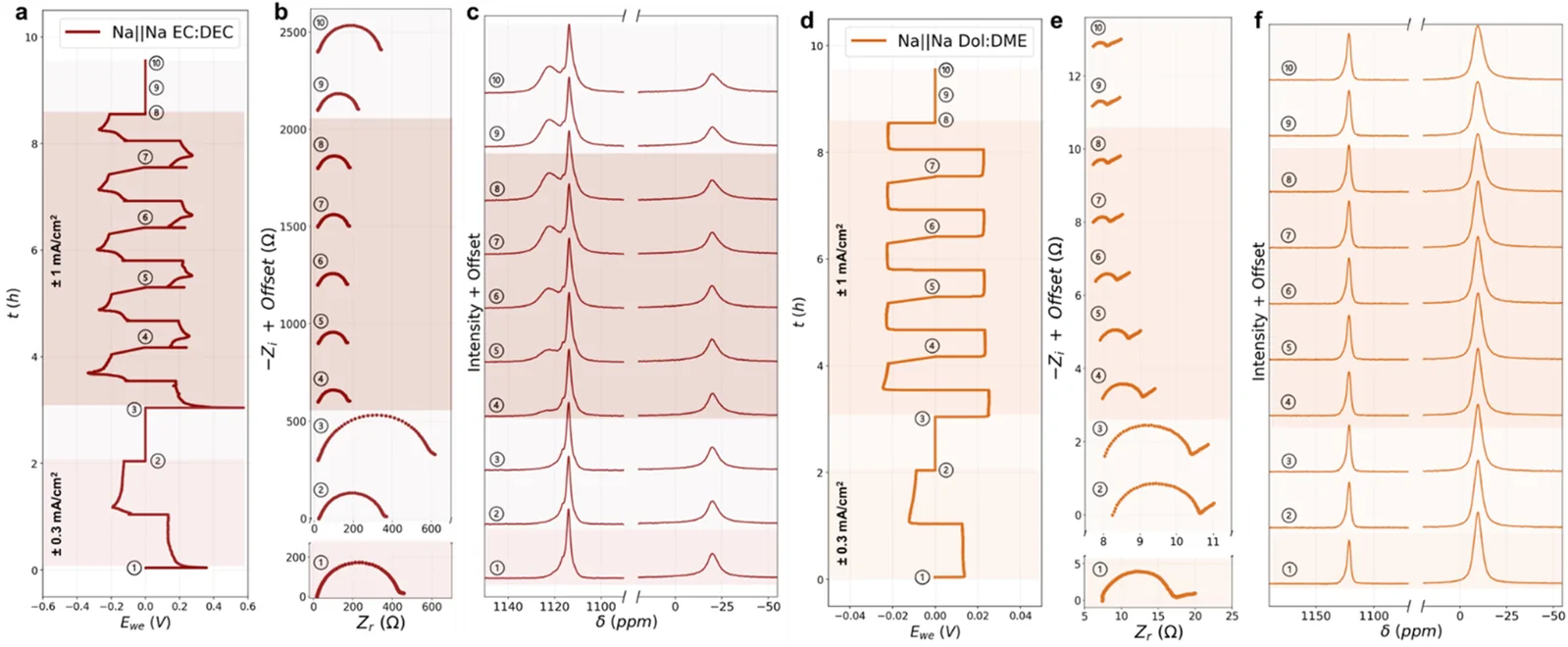
Time-resolved EIS and NMR spectroscopic evolution of symmetric Na‖Na cells in EC:DEC and DOL:DME electrolytes during galvanostatic cycling. (a and d) Voltage profiles recorded during stepwise galvanostatic cycling with an initial formation cycle at current densities of ±0.3 mA cm^−2^ (±0.3 mAh cm^−2^), followed by high-current-density cycles at ±1 mA cm^−2^ (±0.5 mAh cm^−2^) for Na‖Na cells in (a) EC:DEC and (d) DOL:DME. Numbered markers indicate the states at which impedance and spectroscopic measurements were collected. (b and e) Corresponding Nyquist plots obtained at the selected cycling stages, displayed with vertical offsets for clarity. (c and f) *In situ* NMR responses collected at the same states, shown as intensity (offset for clarity) *versus* chemical shift (*δ*, ppm).

For the Li electrode in EC:EMC electrolyte (Fig. 3a–c), the voltage profile during stepwise current cycling shows high polarization as the current density is applied. The Nyquist plots measured at sequential stages reveal pronounced evolution of the semicircle diameter and shape (Fig. 3b). There is a large decrease in the impedance upon formation, which then increases upon resting. Upon cycling with higher current density, the Nyquist plot demonstrates a continuous decrease in size. The corresponding NMR response (Fig. 3c) displays systematic changes in peak intensity and line shape across the same sequence of cycling steps. The spectral features evolve continuously rather than abruptly, indicating progressive modification of the interfacial environment during repeated stripping and plating. The appearance of microstructural Li environments (∼265 ppm) is evident from spectrum 4 onwards, which broadly corresponds to the abrupt reduction of impedance upon the first plating/stripping cycle. This microstructural feature persists and grows in intensity, consistent with the expectation for the EC:EMC electrolyte.

In contrast, the DOL:DME electrolyte system (Fig. 3d–f) exhibits similar behaviour in the formation step but different behaviour upon cycling. The voltage profile shows comparatively lower polarization across the applied current densities (Fig. 3d). The Nyquist plots display smaller semicircles overall and less dramatic reshaping with cycling. While the Nyquist plots show a decrease in size upon cycling, the decrease stabilizes, unlike the continuous decrease observed in the carbonate system (Fig. 3e). The NMR features show a much stronger microstructure signal, evident from the first formation cycle, which again grows in intensity upon repeated plating/stripping cycles, the quantification of which will be discussed in more detail below.

In the Na‖Na EC:DEC electrolyte (Fig. 4), the impedance shows a small decrease after formation. A pronounced voltage polarization at ±1 mA cm^−2^ is accompanied by lower Nyquist responses (Fig. 4a and b). The impedance also increases relative to the pristine state as the cell is rested after low-current cycling. This is an indication of the high chemical reactivity of the newly formed Na microstructure with EC:DEC causing the SEI resistance to increase. This is also observed during the rest period after cycling. During cycling, the Nyquist response remains nearly unchanged between the initial and final cycles (Fig. 4b).

Simultaneously, the NMR response (Fig. 4c) shows progressive growth of a broad metallic resonance centred near ∼1120 ppm, which corresponds to the microstructural metallic Na environments. Consistent with the Li case, this peak first appears after the first plating/stripping cycle (spectrum 4) and gradually grows in intensity. Interestingly, the linewidth and shape of the bulk metallic Na signal (∼1110 ppm) broadens with a pronounced tail toward lower frequency; the line shape resembles disordered second-order quadrupolar broadening of the ^23^Na (*I* = 3/2) nucleus and may indicate increasing structural disorder at the metallic Na surface during cycling. Bulk magnetic susceptibility effects may also cause broadening of this resonance.

In contrast, the DOL:DME system exhibits markedly different behaviour. The voltage profile displays remarkably smaller polarization (≈±0.03 V) compared to the EC:DEC system (≈±0.3 V). The impedance spectra (Fig. 4e) show a continuous decrease in the impedance values with relatively very small values that are associated with a conventional SEI passivating response. The corresponding NMR spectra (Fig. 4f) display an absence of the microstructural metallic Na peak with minimal change across all spectra. Importantly, the square-shaped voltage profile and the unchanged Nyquist response between steps 6–10 in DOL:DME indicate the possible formation of soft shorts. As the formation of soft shorts in this system cannot be ruled out, this observation further supports the dominant role of morphology evolution over SEI chemistry.^8^

The combined EIS and NMR results reveal a clear correlation between impedance magnitude and the microstructural metallic Li/Na signal intensity. Qualitatively, we identify that the increase in the microstructural metallic environment occurs concurrently with the decrease in impedance, which indicates an increase in surface area during cycling, which will be investigated further below.

### Equivalent-circuit fits of EIS and peak analysis for *in situ* NMR

To further quantify the interfacial evolution observed in the time-resolved impedance measurements, the EIS spectra associated with the SEI were fitted using an equivalent-circuit model to extract the SEI resistance (*R*_SEI_) and its associated capacitive response, represented by a constant phase element (CPE) characterized by *Y*_SEI_ and *α*_SEI_. The resistance *R*_SEI_ is attributed to ionic transport through the SEI, while *Y*_SEI_ represents the capacitive contribution associated with the interfacial impedance response.^26,27^ The exponential parameter *α*_SEI_ accounts for the non-ideal capacitive behaviour expected for heterogeneous interfaces and was found to range between 0.8 and 0.9 across all systems.^28,29^ In parallel with the impedance analysis, the spectral peaks observed in the corresponding *in situ* NMR measurements were fitted to quantify structural changes occurring within the cell. Fig. 5 and 6 summarize the evolution of these parameters during the pristine, formation, cycling, and rest stages for Li and Na symmetric cells, respectively.

**Fig. 5:**
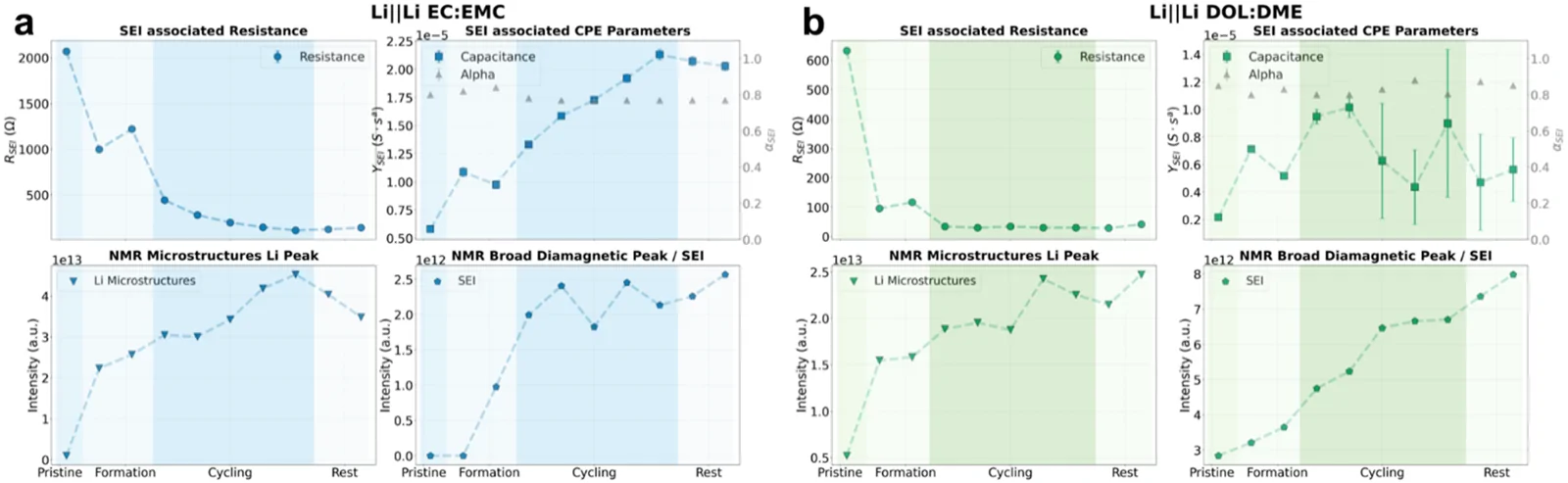
Evolution of SEI resistance and capacitance extracted from equivalent-circuit analysis, and Li microstructure and SEI peak changes observed by NMR during formation, rest, and cycling of symmetric Li cells for (a) Li‖Li in EC:EMC, and (b) Li‖Li in DOL:DME.

**Fig. 6:**
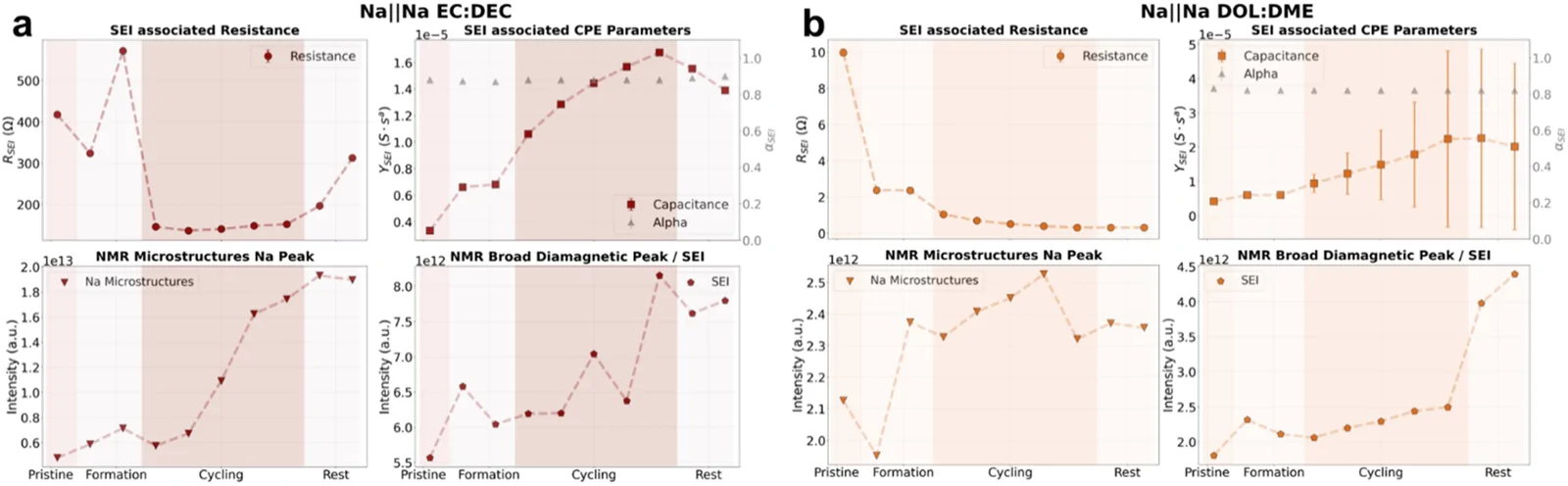
Evolution of SEI resistance and capacitance extracted from equivalent-circuit analysis, and Li microstructure and SEI peak changes observed by NMR during formation, rest, and cycling of symmetric Na cells for (a) Na‖Na in EC:DEC, (b) Na‖Na in DOL:DME.

Upon application of the formation cycle, all systems exhibit a decrease in *R*_SEI_, although the magnitude of this reduction varies between chemistries. The most pronounced decrease is observed in the Li systems, while the Na systems show comparatively smaller absolute changes. Simultaneously, the associated capacitive response (*Y*_SEI_) increases during formation across all systems. Because the capacitive response scales with the electrochemically active surface area, the observed decrease in *R*_SEI_ and increase in *Y*_SEI_ suggest that the EIS response after the formation step is influenced by morphological changes. One interpretation consistent with these trends is an increase in effective surface area arising from microstructural metal formation during the initial plating and stripping processes. The corresponding NMR analysis supports this interpretation, as the microstructure-related peak shows a sudden increase following formation, indicating the development of additional microstructured metallic surface features.

Following the formation step, the cells were allowed to rest under open-circuit conditions. During this period, *R*_SEI_ increases in all systems, although to markedly different extents. The largest increase is observed for Na in EC:DEC, followed by Li in EC:EMC. In contrast, the ether electrolytes show only modest increases for Li and nearly negligible changes for Na. The increase in *R*_SEI_ during rest indicates that the interface continues to evolve even in the absence of applied current. Since no electrochemical cycling occurs during this stage, the resistance growth likely reflects chemically driven interfacial processes rather than morphological changes induced by plating and stripping. However, morphological changes due to the corrosion of high-surface-area metal could potentially drive a decrease in surface area and an apparent increase in *R*_SEI_. The magnitude of the resistance increase therefore suggests different degrees of chemical reactivity between the metal surface—particularly the microstructures formed during formation—and the surrounding electrolyte. This behaviour demonstrates that EIS can sensitively capture the corrosion or chemical evolution of the formed microstructures and the growth of the SEI. Consistent with this observation, the corresponding NMR spectra show an increase in the broad diamagnetic peak associated with SEI components with poor ionic mobility, indicating additional interphase formation during rest. Notably, the capacitive response does not change significantly during this stage, suggesting that the resistance growth is not caused by a significant surface-area change. Instead, the impedance evolution during rest is consistent with continued SEI growth without significant geometric variation of the interface.

Upon subsequent galvanostatic cycling, the evolution of *R*_SEI_ and *Y*_SEI_ differs across the electrolyte–metal systems. In general, changes in resistance are accompanied by variations in the capacitive response, which are commonly interpreted as changes in the electrochemically active surface area during repeated plating and stripping. As microstructures develop, the effective interfacial area increases, which can reduce the apparent resistance normalised to geometric area, while increasing the capacitance extracted from the equivalent-circuit model. Importantly, these geometric effects can mask variations associated with SEI growth or compositional changes, meaning that decreases in *R*_SEI_ should not necessarily be interpreted as an intrinsic improvement in SEI ion transport.

For the Li EC:EMC system, *R*_SEI_ shows a continuous decrease while *Y*_SEI_ progressively increases with cycling. This trend indicates the ongoing formation of metallic microstructures that increase the effective surface area of the electrode. The corresponding NMR measurements support this interpretation, showing a continuous increase in the metallic Li microstructure peak, together with a gradual growth of the SEI-related spectral components. However, the SEI evolution detected by NMR is not reflected in the impedance-derived *R*_SEI_ values, as the continuous increase in surface area dominates the impedance response and masks the contribution of SEI variation.

In the Li DOL:DME system, both *R*_SEI_ and *Y*_SEI_ remain relatively constant throughout cycling, although the capacitance values exhibit larger uncertainties. In contrast, the NMR spectra show a continuous increase in both the microstructure-related Li signal and the SEI-associated components. This behaviour indicates that microstructure formation and SEI accumulation occur during cycling but are not directly captured by the impedance-derived SEI parameters, again highlighting the strong influence of surface-area effects on the EIS response.

For the Na EC:DEC system, *R*_SEI_ remains approximately constant during cycling, while the capacitance evolves progressively. At the same time, the NMR measurements show increasing signals associated with both Na microstructures and SEI components. The simultaneous evolution of capacitance and NMR features suggests that both surface-area growth and continued SEI accumulation occur during cycling. However, the constant *R*_SEI_ indicates that these processes cannot be clearly resolved by EIS alone, as the influence of increasing surface area obscures changes related to SEI thickness or composition.

In contrast, the Na DOL:DME system exhibits very small *R*_SEI_ values, indicating minimal resistance to Na^+^ transport across the interface. Only a slight increase in capacitance is observed during cycling. The corresponding NMR spectra show very limited formation of Na microstructures and SEI components, consistent with the formation of a thin and highly conductive SEI. This behaviour suggests that the low interfacial resistance enables relatively homogeneous Na plating, which suppresses extensive microstructure formation. Alternatively, the limited formation of Na microstructures may suggest the occurrence of soft shorts; however, this possibility is beyond the scope of the present study.

### Temperature-dependent EIS analysis

Temperature-dependent EIS provides an additional dimension for probing the SEI properties. By examining the variation of impedance parameters extracted from equivalent-circuit fits (*R*_SEI_ and *Y*_SEI_) as a function of temperature, it is possible to extract activation energies associated with the probed process and gain insight into the thermally activated nature of SEI ionic transport.^30–33^Fig. 7 presents Arrhenius plots derived from the equivalent-circuit analysis for both the SEI resistance (*R*_SEI_) and the corresponding time constant (*τ*_SEI_) for Li and Na symmetric cells in carbonate and ether electrolytes. The time constant (*τ*_SEI_) is obtained by multiplying *R*_SEI_ and *Y*_SEI_. We compare the variation in activation energy calculation between *R*_SEI_ and *τ*_SEI_ since *R*_SEI_ can be influenced by the changes in the surface area shown previously and indicated by other studies.^19^

**Fig. 7:**
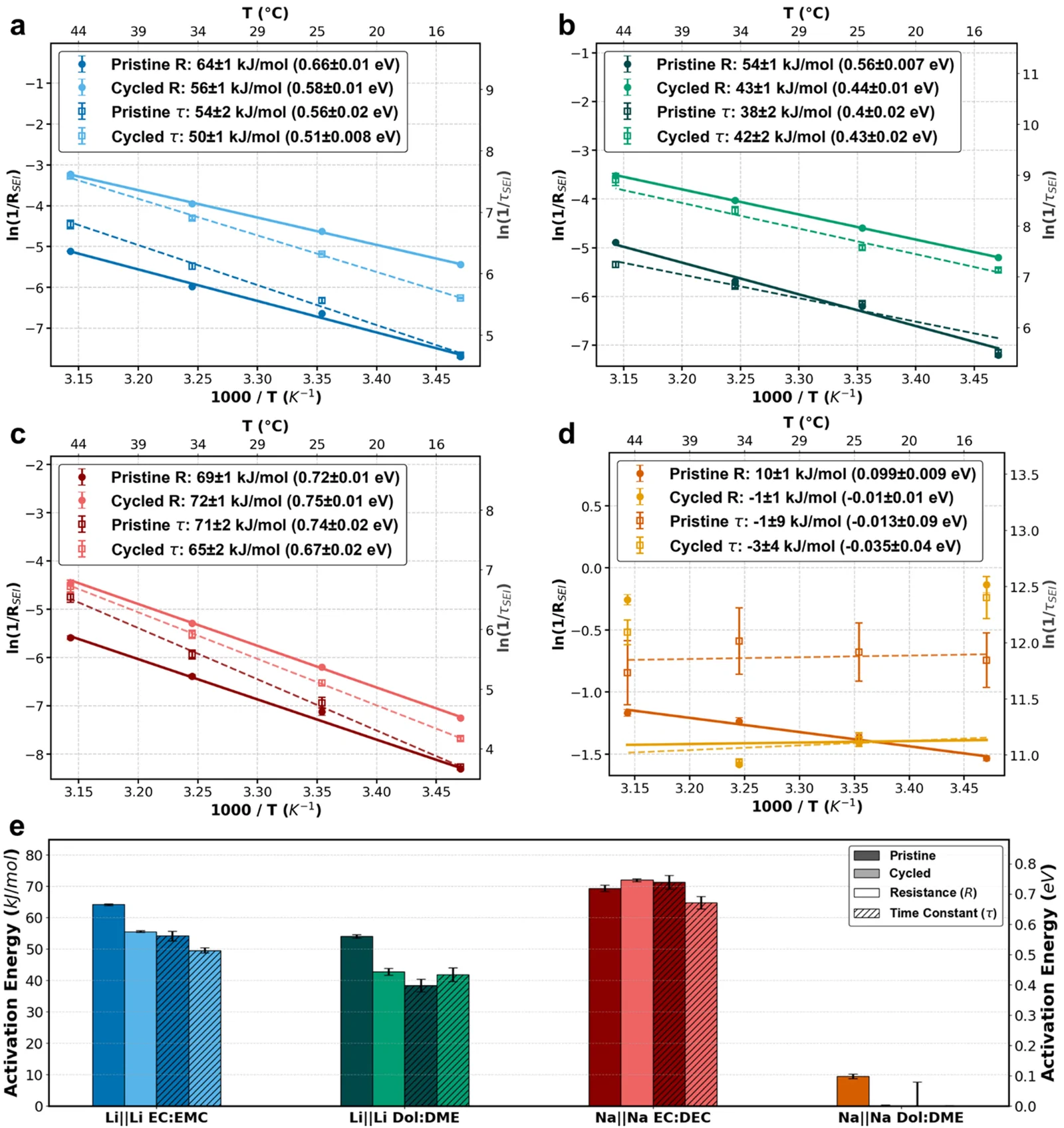
Temperature-dependent impedance analysis and extraction of activation energies for SEI-related processes in Li and Na symmetric cells. Arrhenius plots of ln(1/*R*_SEI_) (left axes) and ln(1/*τ*_SEI_) (right axes) as a function of 1000/*T* for (a) Li‖Li in EC:EMC, (b) Li‖Li in DOL:DME, (c) Na‖Na in EC:DEC, and (d) Na‖Na in DOL:DME. Solid and dashed lines represent pristine and cycled states, respectively. Activation energies extracted from linear fits are reported in each panel for both resistance-derived and time-constant-derived parameters. (e) Summary of activation energies for all systems, comparing pristine and cycled states and distinguishing values obtained from resistance (*R*) and the time constant (*τ*). While resistance-derived activation energies reflect the apparent temperature dependence of interfacial impedance, the time constant incorporates both resistive and capacitive contributions, reducing surface-area dependence and more directly probing intrinsic interphase kinetics.

The natural logarithm of the inverse resistance, ln(1/*R*_SEI_), plotted as a function of 1000/*T*, shows linear behavior across the investigated temperature range for all systems (Fig. 7a–d). This linearity indicates that the extracted resistance follows Arrhenius-type temperature dependence, allowing activation energies to be determined from the slope. For Li in EC:EMC, the pristine SEI exhibits an activation energy of ∼64 kJ mol^−1^, which decreases after cycling to ∼56 kJ mol^−1^. A similar reduction is observed for Li in DOL:DME, where the activation energy decreases from ∼54 to ∼43 kJ mol^−1^ after cycling. These reductions suggest that cycling modifies the thermally activated transport characteristics of the interface.

In the Na systems, different trends emerge. For Na in EC:DEC, the activation energy associated with resistance slightly increases after cycling (from ∼69 to ∼72 kJ mol^−1^). In contrast, Na in DOL:DME exhibits very small apparent activation energies, with values near zero within experimental uncertainty, suggesting minimal temperature dependence of the extracted resistance in this configuration.

While resistance-based activation energies provide useful information, it is important to recognize that *R*_SEI_ is inherently influenced by the electrochemically active surface area. Any temperature-dependent morphological changes or pre-existing surface roughness can alter the absolute resistance values, potentially affecting the extracted activation energy. To address this limitation, we also examined the temperature dependence of the characteristic time constant, *τ*_SEI_. The time constant incorporates both resistive and capacitive contributions and thus reduces explicit surface-area dependence. Because capacitance scales with surface area in a manner opposite to resistance, their product (which defines the time constant) minimizes geometric contributions and better reflects the intrinsic interfacial kinetics.

The Arrhenius plots of ln(1/*τ*_SEI_) reveal trends that differ in magnitude from those obtained using resistance alone. For Li systems, the activation energies derived from *τ*_SEI_ are consistently slightly lower than those extracted from resistance, and they also decrease upon cycling. In Na EC:DEC, the time-constant-based activation energy decreases after cycling, in contrast to the resistance-based value. For Na DOL:DME, the activation energies derived from *τ*_SEI_ remain very small and close to zero, consistent with weak temperature sensitivity. The relatively large errors observed for Na in DOL:DME highlight the limitations of the method in capturing SEI evolution when the SEI deviates substantially from classical behavior.

The comparison between resistance-derived and time-constant-derived activation energies highlights an important methodological point: resistance captures both intrinsic transport barriers and geometric contributions, whereas the time constant more directly reflects the intrinsic kinetic properties of the SEI. Differences between the two extracted activation energies therefore provide insight into the relative contributions of surface-area evolution *versus* intrinsic SEI transport properties.

Fig. 7e summarizes the extracted activation energies and highlights clear electrolyte- and metal-dependent differences. Carbonate systems in both Li and Na exhibit higher activation energies than ether systems, indicating stronger temperature dependence of interfacial transport. Upon cycling, the activation energies in carbonate electrolytes decrease, suggesting a reduction in the effective transport barrier, consistent with increased interfacial porosity and improved ion pathways. In contrast, Li in the ether electrolyte shows an increase in activation energy after cycling, indicating a higher apparent transport barrier and possible interphase growth or densification. For Na in the ether electrolyte, the activation energy remains negligible, consistent with the weak temperature dependence of its low interfacial resistance and indicative of a thin, highly conductive SEI.

These variations can be attributed to differences in SEI composition arising from the choice of electrolyte. The ionic transport properties of the SEI are strongly governed by the relative contributions of inorganic phases (*e.g.*, LiF, Li_2_CO_3_, Li_2_O) and organic or polymeric species (*e.g.*, ROCO_2_Li, oligocarbonates).^34,35^ Inorganic-rich SEIs tend to be dense and highly ordered, where ion transport proceeds predominantly through defect-mediated hopping or along interfacial pathways, typically resulting in higher interfacial resistance and larger activation energies (*E*_a_ ≈ 0.6–0.8 eV).^36,37^ In contrast, organic-rich SEIs are generally more porous and solvent-swollen, enabling transport mechanisms closer to liquid-like diffusion or segmental motion, which leads to lower activation energies (*E*_a_ ≈ 0.2–0.4 eV) and reduced resistance.^38,39^ These distinctions are further amplified in Na systems, where the larger ionic radius of Na^+^ imposes additional transport limitations, particularly within dense inorganic phases.^40,41^ Consistent with these trends, our results show lower activation barriers for ether-based electrolytes compared to carbonate-based systems, indicating the formation of more organic, transport-facilitating SEI layers. In contrast, the higher activation energies observed in Na systems point to more hindered ion transport across the interphase, in agreement with their more resistive SEI characteristics.

Overall, the combined analysis highlights both the capabilities and the limitations of EIS for probing SEI behaviour in symmetric Li and Na systems. EIS is effective in tracking changes in interfacial resistance and capacitance during formation, cycling, and rest, providing valuable insight into the evolution of the electrode interface and the degree of surface-area variation occurring during metal deposition and stripping. In particular, variations in *R*_SEI_ and *Y*_SEI_ can sensitively capture processes such as electrochemical corrosion of the formed metal microstructure during rest and the growth of the SEI. However, the results also demonstrate that impedance-derived SEI parameters are strongly influenced by geometric effects associated with microstructure formation. As the surface area of the electrode evolves, the apparent resistance and capacitance can change independently of intrinsic SEI properties, masking variations in SEI composition or thickness. Consequently, decreases in *R*_SEI_ should not be interpreted directly as improved ion transport through the interphase. The comparison with *in situ* NMR measurements clearly shows that significant microstructure growth and SEI accumulation can occur without being fully reflected in the impedance response. Therefore, while EIS provides a powerful tool for monitoring interfacial evolution, it cannot independently resolve SEI growth or structure evolution in symmetric Li- and Na-metal systems without complementary techniques that can directly probe structural and chemical changes at the interface.

## Conclusions

This work evaluates the strengths and limitations of EIS as a tool for characterizing the SEI in Li- and Na-metal cells using carbonate and ether electrolytes. By combining time-resolved EIS, DRT analysis, equivalent-circuit modelling, temperature-dependent measurements, and *in situ* NMR, the evolution of interfacial resistance and capacitance was tracked during formation, cycling, and rest. The results show that EIS effectively captures interfacial changes such as corrosion of the formed microstructures during rest and surface-area growth during cycling.

However, the study also demonstrates that impedance-derived SEI parameters are influenced by both electrode surface-area changes associated with microstructure formation and SEI formation. These two factors are difficult to decouple by simply evaluating the resistance and capacitance in EIS measurements: variations in surface morphology can mask changes in SEI thickness or composition. Comparison with *in situ* NMR results confirms that significant microstructure growth and SEI accumulation can occur without being clearly reflected in the impedance response. Temperature-dependent analysis further shows that activation energies derived from resistance can include geometric contributions.

Overall, we conclude that while EIS provides valuable insight into interfacial evolution, it cannot independently resolve SEI growth or structural evolution in symmetric alkali-metal systems. Therefore, reliable interpretation of EIS data for selectively probing SEI phenomena requires the deconvolution of effects due to changes in surface morphology from those of the SEIs; to this end, we suggest that impedance measurements should be combined with complementary characterization techniques such as *in situ* NMR. By correlating these independent yet complementary datasets, it becomes possible to more reliably decouple morphology-driven changes from intrinsic SEI-related contributions in the EIS response, leading to a more accurate and physically grounded interpretation of interfacial processes. Future work will further extend this approach by developing NMR techniques to allow quantitative analysis of dendritic Li/Na formation and linking these morphological features to changes in effective surface area, alongside the combined quantitative and qualitative assessment of SEI components.

## Conflicts of interest

There are no conflicts to declare.

## Data Availability

Data for this article are available at the Apollo repository: https://doi.org/10.17863/CAM.129485.
